# A Rare Occurrence of Three Primary Malignancies of the Rectum, Breast, and Kidney in the Same Patient: A Case Report and Review of the Literature

**DOI:** 10.1155/2019/1716029

**Published:** 2019-09-09

**Authors:** Umesh Jayarajah, Oshan Basnayake, Pradeep Wijerathne, Jayan Jayasinghe, Dharmabandhu N. Samarasekera, Sanjeewa Seneviratne

**Affiliations:** ^1^Professorial Surgical Unit, National Hospital of Sri Lanka, Colombo, Sri Lanka; ^2^Department of Surgery, Faculty of Medicine, University of Colombo, Sri Lanka

## Abstract

An increasing number of patients with multiple primary cancers are encountered due to improved cancer detection, widespread cancer screening, and better cancer treatment. Here, we report such a patient without a family history of malignancies or a known genetic predisposition developing three primary malignancies of the rectum, breast, and kidney. A 63-year-old female who underwent an anterior resection for rectal cancer was detected to have an elevated carcinoembryonic antigen (CEA) level during a routine follow-up, 8 years after the initial surgery. Clinical examination revealed a left breast lump which was confirmed as invasive ductal carcinoma (pT3 pN0 cM0). Imaging and colonoscopy excluded a local recurrence. However, a right renal lesion suggestive of a renal cell carcinoma was detected (pT1a). She underwent left mastectomy with a sentinel lymph node biopsy and a right partial nephrectomy with a curative intent. Postoperatively, CEA levels declined to normal limits. Management of multiple primary malignancies poses a major challenge. A multidisciplinary approach and tailored decision-making for the individual patient help with the optimum outcome.

## 1. Introduction

Occurrence of multiple primary malignancies in an individual is a rare phenomenon.

Due to the combined effect of better detection, screening, improved cancer treatment modalities, and prolonged life expectancy, more and more individuals with multiple primary cancers are encountered [1]. The occurrence of multiple primary tumours, either synchronous or metachronous, poses significant challenges in the management of such patients including the type, timing, and sequence of treatments.

The prevalence of multiple primary tumours is estimated between 0.7% and 11.7%, and the occurrence is higher with increasing age [1]. Patients with a history of malignancy are at a 14% higher risk of developing a second primary malignancy compared with the general population [2]. Furthermore, females have been shown to have a higher risk than males in developing multiple primary tumours. The most common sites associated with multiple primaries include the colon, breast, lung, and skin melanoma [2].

Although the incidence is low, the association of two different primary cancers in a single patient has been widely reported. However, the development of more than two primary unrelated malignancies is still considered a rare phenomenon, especially in the absence of a family history or specific genetic predisposition. This case reports such a patient without a family history of malignancies or a known genetic predisposition developing three primary malignancies of the rectum, breast, and kidney.

## 2. Case Presentation

A 63-year-old female with a history of diabetes mellitus, hypertension, and dyslipidaemia underwent a curative low anterior resection combined with neoadjuvant chemoradiotherapy and adjuvant chemotherapy for a moderately differentiated adenocarcinoma of the rectum. She had an uncomplicated postoperative period and good response to the neoadjuvant and adjuvant therapies. She was under surveillance with colonoscopies and annual carcinoembryonic antigen (CEA) levels for 8 years without any evidence of recurrence.

During a routine follow-up 8 years after surgery for rectal cancer, she was detected to have an elevated CEA level (17 ng/ml). She was completely asymptomatic without any clinical evidence of local or metastatic recurrence. On examination, she was found to have a clinically malignant 5 cm lump in the left breast with no palpable ipsilateral axillary lymphadenopathy. Her abdominal and digital rectal examination was unremarkable. Her basic biochemical investigations were within normal limits.

**Figure 1:**
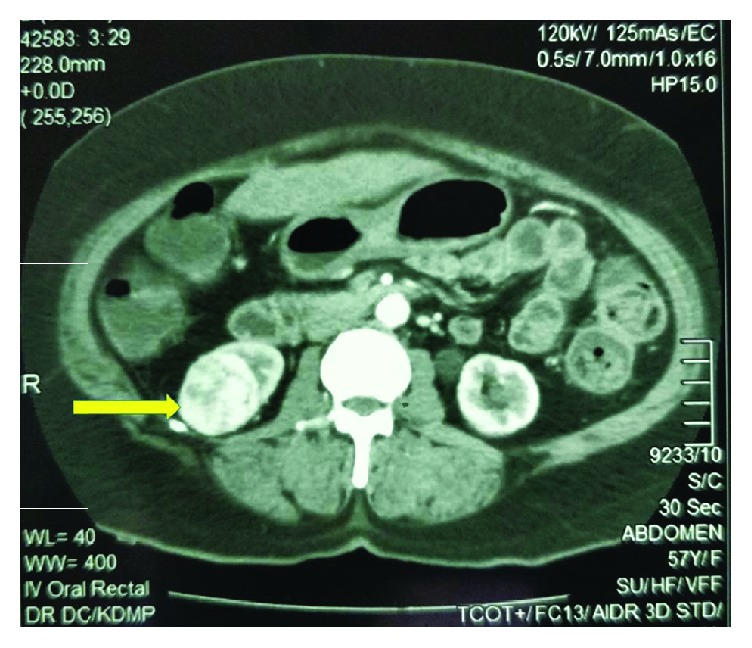
A heterogeneously enhancing well-defined nodule in the lower pole of the right kidney measuring 4 × 4.1 × 4.6 cm with no evidence of perilesional organ invasion or lymph node involvement, suggestive of an early neoplastic lesion.

To evaluate for the rise in CEA levels, she underwent a colonoscopy which showed no evidence of tumour recurrence or metachronous neoplastic lesions in the rest of the colon. Contrast-enhanced computed tomography (CECT), however, showed a heterogeneously enhancing well-defined nodule in the lower pole of the right kidney measuring 4 cm with no evidence of perilesional organ invasion or lymph node involvement, suggestive of an early renal cell carcinoma (Figure 1). Furthermore, there was an enhancing well-defined area in the lower lobe of the right lung measuring 1.5 cm which was indeterminate in nature. She underwent a CT-guided biopsy of the lung lesion which showed no evidence of a primary or secondary neoplasm.

**Figure 2:**
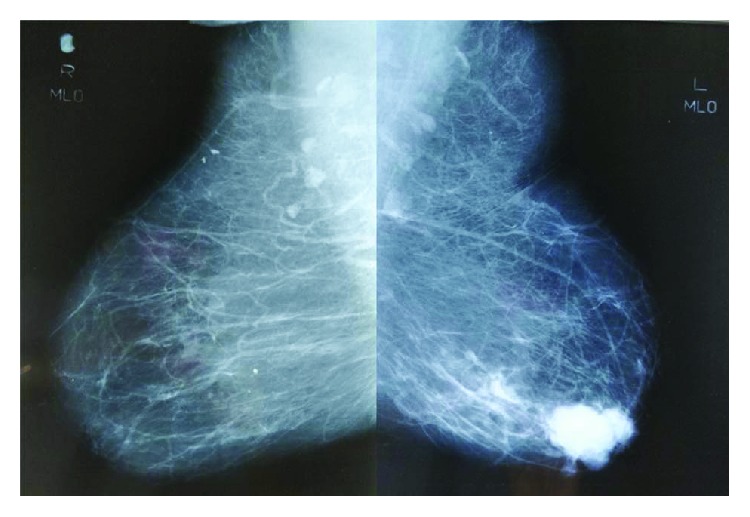
Bilateral mediolateral-oblique mammogram images showing a lobulated, well-defined, high soft tissue density mass in the left outer quadrant, 1 cm away from the nipple with an associated irregular soft tissue density tract seen extending posteriorly. Benign appearance of the right breast.

Mammography and ultrasonography of the breasts showed a 5.0 cm lobulated, well-defined, high soft tissue density mass in the left outer quadrant, 1 cm away from the nipple with an associated irregular soft tissue density tract seen extending posteriorly and peripherally into the left outer quadrant from the mass (Figure 2). Ultrasound showed a lobulated hypoechoic mass and further 2 oval-shaped hypoechoic solid nodules 3.5 cm away from it. These findings were highly suggestive of a malignancy (BIRADS V) of the breast. There was no suspicious axillary lymphadenopathy. A core biopsy of the lump confirmed an invasive ductal carcinoma.

A multidisciplinary team discussion was conducted among the general surgeon, gastrointestinal surgeon, urologist, radiologist, histopathologist, and oncologist. An agreement was reached based on the findings that the three tumours were unrelated. A decision was reached to perform the mastectomy and partial nephrectomy at the same setting and provide adjuvant chemotherapy.

The patient underwent a left mastectomy with a sentinel lymph node biopsy and right lower pole partial nephrectomy during a combined operation. The frozen section of sentinel lymph nodes was negative, and therefore, axillary lymph node dissection was not performed. Postoperative recovery was uneventful. Histology of the breast tumour revealed a 4.5 cm invasive ductal carcinoma of the breast (no special type (NST)) with minimal tubule formation (<5%—score 3/3), nuclear grade of III (score 3/3), and mitotic count of 4/10 (score 1/3) which was compatible with modified Nottingham grade II. The adjacent area showed a tumour of similar morphology with a predominantly high grade, solid, ductal carcinoma in situ (DCIS) component involving 70% of the area. Vascular or perineural invasion was not seen. The four sentinel lymph nodes were confirmed to have reactive changes only. Immunohistochemistry showed ER and PR positivity, and Her2/neu was negative (molecular subtype luminal A). The Ki 67 proliferative index was 15%. TNM staging was pT3 pN0 cM0.

Histology of the partial nephrectomy showed a clear cell renal cell carcinoma with a predominantly alveolar growth pattern. The constituent cells had abundant clear cytoplasm and central nuclei with inconspicuous nucleoli (WHO/ISUP grade 1). Microvascular or perineural invasion was not seen. The surgical resection margin was negative although there was a focal area where it was less than 0.1 mm. The tumour was confined within the renal capsule, and the surrounding fat was not invaded by the tumour. The pathological tumour stage was pT1a. After a multidisciplinary team discussion with the surgeons, pathologist, and oncologist, a decision was made to start her on tamoxifen as adjuvant therapy for breast cancer with no adjuvant therapy for the renal cell carcinoma. She tolerated the treatment well and did not experience any adverse effects.

A follow-up CT scan of the chest was done in 6 months which showed no progression of the lung nodule. CEA levels declined to normal values postoperatively.

## 3. Discussion

Multiple primary cancers are defined as two or more cancers occurring in the same patient with no subordinate relationship [3]. These may occur as synchronous and metachronous cancers [3]. Theodor Bilroth was the first to report multiple primary malignancies in a single patient in 1879. To qualify to be multiple primaries, he thought that tumour should have a distinct histology, arise from a different organ, and should have the ability to produce its own metastasis. These criteria were modified later and included the term that “possibility of one being a metastasis of another must be excluded” [4].

Double primary cancers are the commonest which are seen in up to 11% cancer patients [1, 3]. Triple primary cancers occur in <0.5% of patients with cancer, and quadruple primary cancers are even rare with a reported rate of <0.1% [5]. In a study by Wu et al. including 1,311 patients with colorectal cancer, 59 patients had multiple primary cancers. Of these patients, 2 (3.39%) had triple cancer and 1 (1.69%) had quadruple cancer. However, the majority of these were synchronous or metachronous colorectal cancers [6]. In another similar review of 308 cases of colorectal carcinoma, 12 cases of colorectal multiple primary cancers and 14 cases of colorectal primary cancers associated with extracolonic primary cancers were detected. Of those, only two patients had three primary malignancies [7].

In a retrospective study from Southeast England, a total of 127,281 patients with primary colorectal cancers between 1961 and 1995 were analysed to look for subsequent occurrence of recurrence or new cancers. In that analysis, women with colorectal cancer before the age of 65 years had a significantly higher risk of developing a subsequent colon cancer as well as cancers of the uterine cervix and ovary [8]. In a Japanese study by Kato et al., multiple primary cancers at extracolonic sites were diagnosed in 117 of 1,111 primary colorectal cancer patients. Of those, gastric cancer was the commonest second cancer (44%). In that cohort, only nine patients (0.01%) had triple primary cancers [9].

The aetiology of multiple primary cancer is complex, and several theories have been proposed. These include a combination of factors such as genetic predisposition, environmental factors, and hormonal factors [10]. Furthermore, treatment-related factors such as exposure to chemotherapy and radiotherapy can predispose an individual to develop future cancers.

A study by Iioka et al. [11] found that patients with multiple primary cancers have a high occurrence of microsatellite instability, stating this as a possible pathophysiological mechanism. Furthermore, multiple primary cancers are also described in patients who have Li-Fraumeni syndrome, p53 tumour suppressor gene mutations, von Hippel-Lindau syndrome, and multiple endocrine neoplasia [12]. Such patients usually have a family history of malignancy and tend to develop specific types of malignancies. To date, no association has been described among the three malignancies reported in this patient.

Our patient did not have a family history of malignancy, and therefore, genetic testing was not done as it was deemed unnecessary following genetic counselling. Considering available evidence for the aetiology for multiple cancers, it is likely to be multifactorial with possible influences from the environment and previous cancer therapy and with likely minimal genetic influence. The patient was initially evaluated following a rise in CEA levels, suspecting a recurrence or a metachronous large bowel cancer. However, colonoscopy and CECT did not show evidence of a bowel tumour. CEA is a nonspecific tumour marker, and the levels are known to be elevated in both breast cancer and a subset of renal cell cancers [13, 14]. Her follow-up CEA was normal indicating that the rise in CEA was due to one or both of these malignancies.

There are no established therapeutic guidelines for multiple primary cancers. Prospective studies are required to analyse magnitude of the problem and study the predisposing factors. Currently, there are no large-scale prospective studies analysing the treatment and outcomes of patients with multiple primary cancers. Therefore, treatment is tailored to the individual patient based on the type of cancer, tumour biology, staging, response to therapy, and patient's general condition [3]. If each of these cancers has the possibility for a cure, radical therapy is generally indicated.

## 4. Conclusions

Our case report describes a triple primary malignancy in a patient who subsequently had successful treatment with the potential for a cure. As triple primary malignancies are rare, there are no large case studies describing the characteristics and outcomes in such patients. Management of these patient poses several challenges, and a multidisciplinary approach with tailored decision-making for the individual patient helps to achieve optimum outcomes.
